# Supplementary material for: The Loroxanthin Cycle: A New Type of Xanthophyll Cycle in Green Algae (Chlorophyta)
Source: Front Plant Sci. 2022 Feb 17;13:797294. doi: 10.3389/fpls.2022.797294 (PMC8891138; doi:10.3389/fpls.2022.797294)
Supplement: Supplementary file 1 [file Data_Sheet_1.docx]

## Supporting Information

Article title: The loroxanthin cycle: A new type of xanthophyll cycle in green algae (*Chlorophyta*)

Authors: Tomas E van den Berg and Roberta Croce.

The following Supporting Information is available for this article:


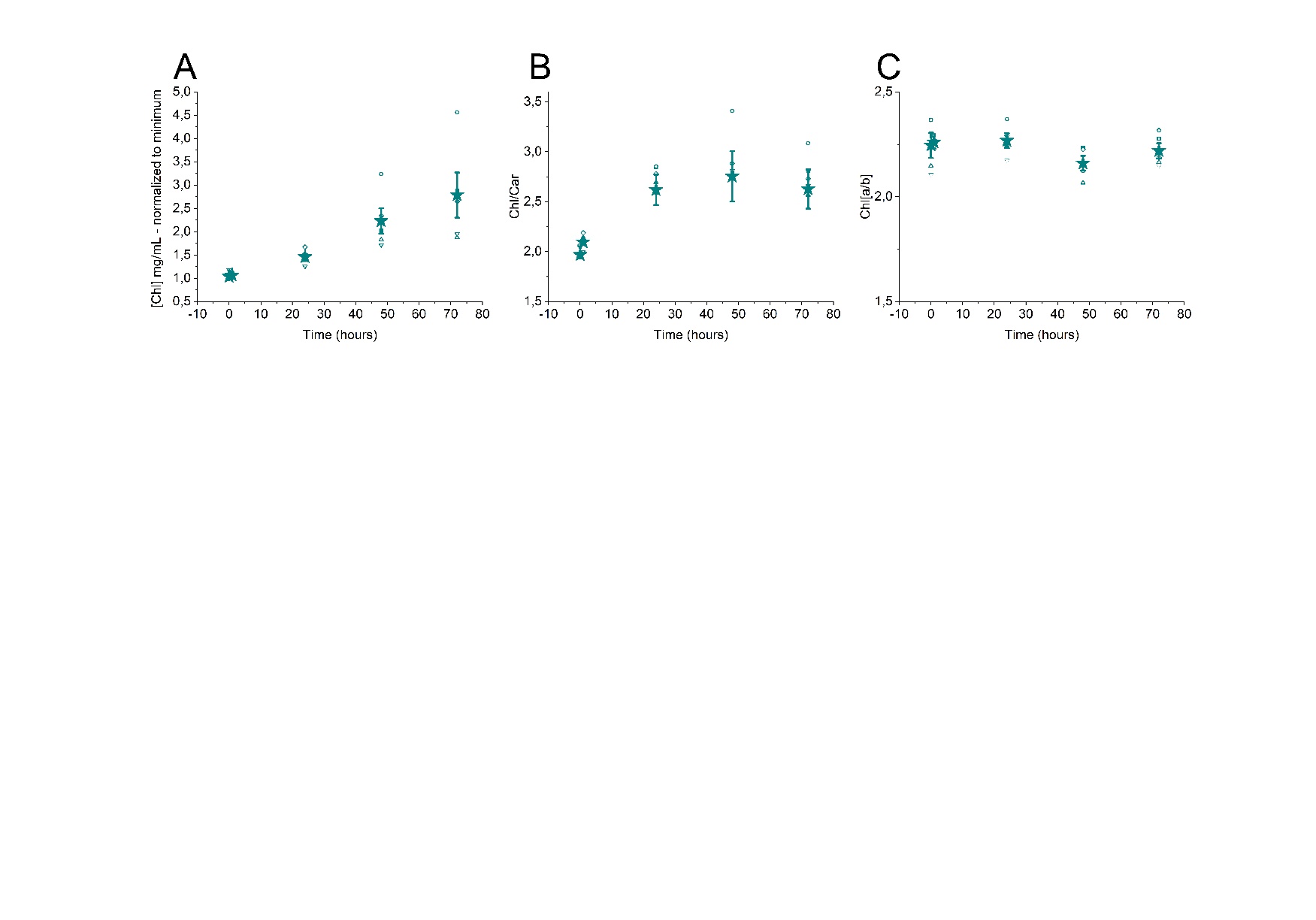


**Fig. S1** **Time course of cellular Chl content (mg/mL) (A). Chl/Car ratio (mol / mol) (B) and Chl *a*/*b* ratio (mol / mol) (C) of the five biological replicates following the shift from HL to LL.** Chl/Car is significantly different from 0hrs (HL) from 1 hour LL onwards (P<0.05). Four technical replicates per biological replicate. Star symbols represent the average. The error bar represents the standard error of the mean.

**Table S1 Students paired one-tailed t-test to determine significance of the changes between the different timepoints in figure 3. (A)** Lo/(L+Lo) Fig. 3C **(B).** ((0.5*A)+Z)/(V+A+Z) Fig. 3B. P values below 0.05 level are indicated by a black number and above by a red number. * indicate that data from one biological replicate for the protocol that separates neoxanthin and loroxanthin (see M&M) is missing due to loss of sample (t=18) while replicates are available for this time point for all other pigments and for the sum Neoxanthin + Loroxanthin.

Lo/(L+Lo)

| **A** | **t(0)** | **t(3)** | **t(6)** | **t(9)** | **t(12)** | **t(15)** | **t(18)** |
| --- | --- | --- | --- | --- | --- | --- | --- |
| t(3) | 0.19 |  |  |  |  |  |  |
| t(6) | 0.43 | 0.01 |  |  |  |  |  |
| t(9) | 0.12 | 0.01 | 0.01 |  |  |  |  |
| t(12) | 0.08 | 0.01 | 0.01 | 0.05 |  |  |  |
| t(15) | 0.48 | 0.02 | 0.34 | 0.04 | 0.04 |  |  |
| t(18) | * | * | * | * | * | * |  |
| t(24) | 0.14 | 0.15 | 0.06 | 0.03 | 0.03 | 0.04 | * |

((0.5*A)+Z)/(V+A+Z)

| **B** | **t(0)** | **t(3)** | **t(6)** | **t(9)** | **t(12)** | **t(15)** | **t(18)** |
| --- | --- | --- | --- | --- | --- | --- | --- |
| t(3) | 0.01 |  |  |  |  |  |  |
| t(6) | 0.05 | 0.08 |  |  |  |  |  |
| t(9) | 0.02 | 0.02 | 0.16 |  |  |  |  |
| t(12) | 0.03 | 0.10 | 0.08 | 0.00 |  |  |  |
| t(15) | 0.03 | 0.01 | 0.01 | 0.00 | 0.00 |  |  |
| t(18) | 0.24 | 0.02 | 0.03 | 0.00 | 0.00 | 0.02 |  |
| t(24) | 0.28 | 0.01 | 0.00 | 0.00 | 0.01 | 0.01 | 0.46 |


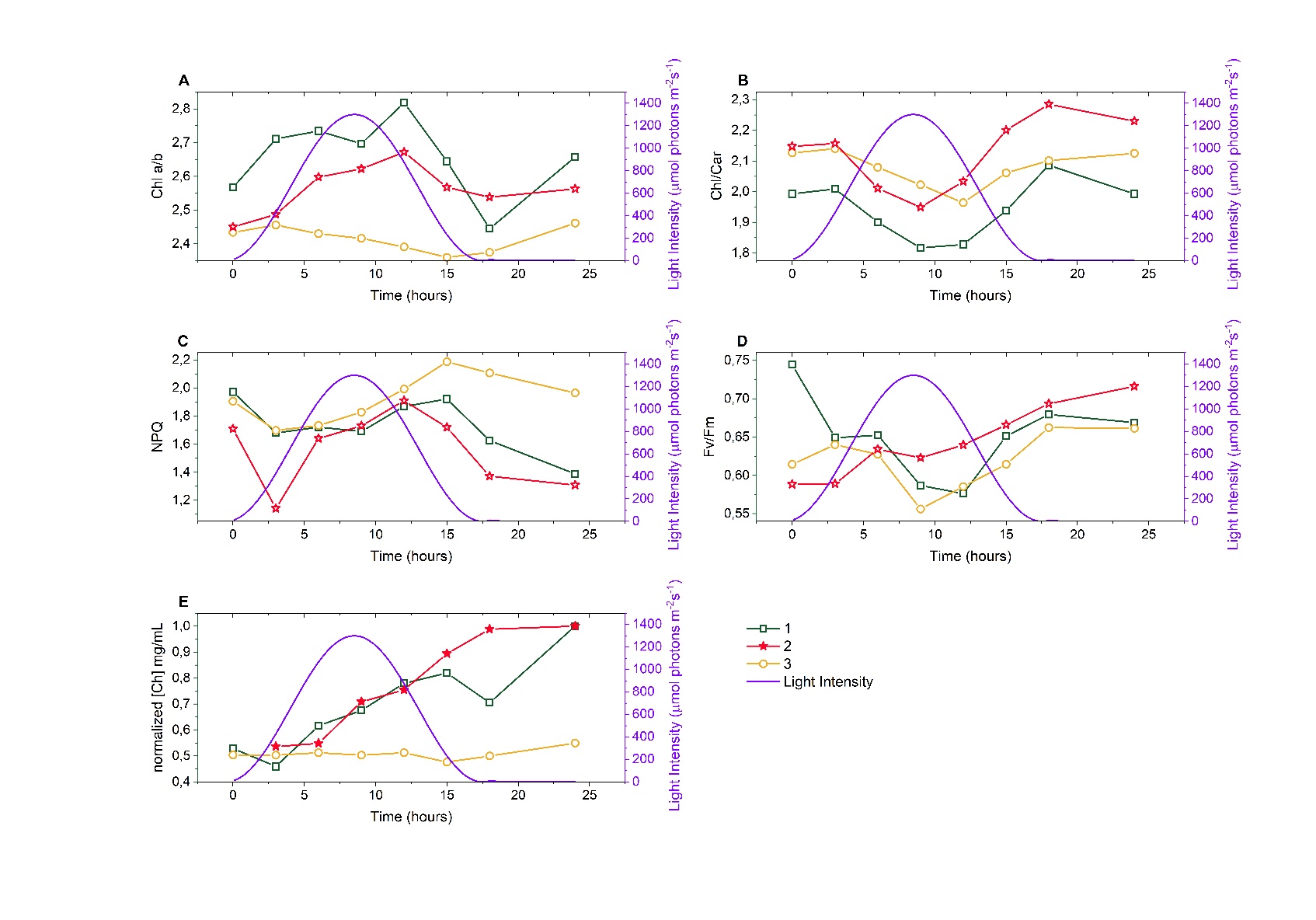


**Fig. S2 Changes in C. reinhardtii cell culture during a “simulated summer” day (18:6 (D:N) sinusoidal light regime)** (**A**) Chl *a*/*b* (mol / mol) (**B**), Chl/car (mol / mol) (**C,D**) Cells were taken from the culture and dark-adapted for twenty minutes in a quartz cuvet while stirring in a Dual-PAM. F_m_ and F_0_ were determined and then an induction curve was taken for 15 min with 2080 µmol photons m^-2^ s^-1^ actinic light. F’ and F_m_’ were measured every 3 min and F_v_/F_m_ and NPQ were calculated as: $\frac{\mathrm{Fv}}{\mathrm{Fm}}=\frac{\mathrm{Fm}^{'}-F}{\mathrm{Fm}^{'}}$and $NPQ= \frac{Fm- Fm'}{\mathrm{Fm}'}$ . Final (after 15 min) NPQ (**C**) and F_v_/F_m_ (**D**) values were plotted. (**E**) [Chl] in mg/ml during 24 hours of cultivation of three biological replicates normalized tot the maximum. Biological replicate 3 deviated from biological replicate 1 and 2 because the Chlorophyll concentration and Chl a/b of the culture did not change during the 24 hours of measurements. We suspect that replicate three was perhaps diluted too much and therefore experiencing a lag phase in growth, leading to a lack of increase of the Chl concentration

**Table S2 Cellular pigment composition of some of the fully acclimated “high” and “low” light cells used for the purification of LHCII.** Xanthophyll content is normalized to 100 Chls (a+b) molecules. n indicates the number of biological replicates. Errors represent the standard error of the mean. N (Neoxanthin), V (Violaxanthin), A (Antheraxanthin), Z (Zeaxanthin), Lo (Loroxanthin), L (lutein), β-Car (β-Carotene).

|  | **N** | **V** | **A** | **Z** | **Lo** | **L** | **β-Car** |
| --- | --- | --- | --- | --- | --- | --- | --- |
| **HL-acclimated**  **(500 µmol photons m^2^s^-1^) (n=5)** | 3.8±0.1 | 3.8±0.2 | 2.0±0.3 | 2.4±0.3 | 0.5±0.2 | 21±1 | 10±2 |
| **LL-acclimated (15 µmol photons m^2^s^-1^) (n=2)** | 5.75±0.01 | 4.233±0.004 | 0 | 0 | 7.50±0.02 | 7.7±0.4 | 5.45±0.02 |

**
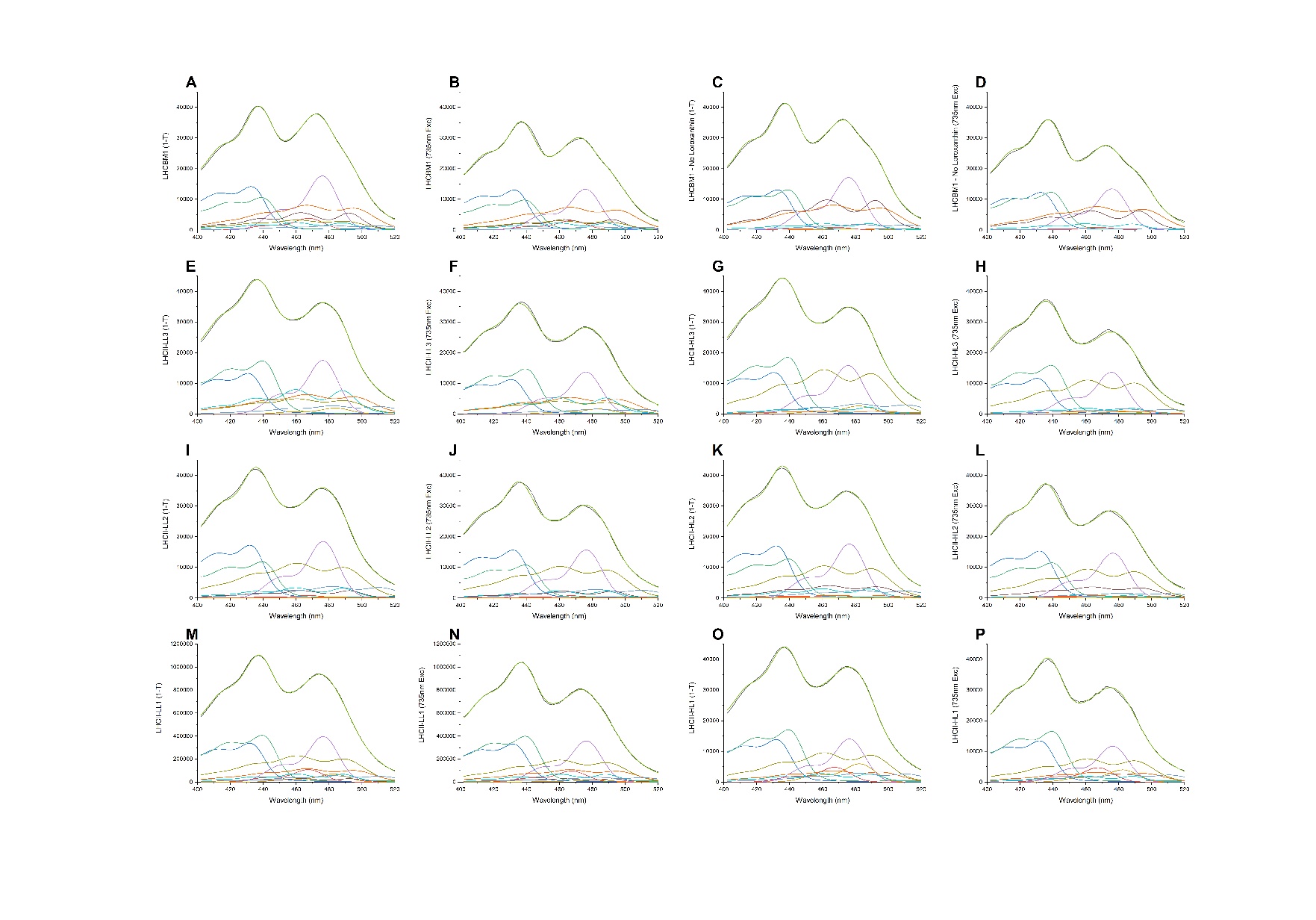
**

**Fig. S3 Fitting of 1-Transmission (1-T) and Fluorescence excitation spectra to determine Car -> Chl  *a* Excitation Energy Transfer efficiency.** LHCBM1 (1-T) **A**, 735 nm excitation **B,** LHCBM1 – No loroxanthin) (1-T) **C**, 735 nm excitation **D,** LHCII-LL3 (1-T) **E**, 735 nm excitation **F**, LHCII-HL3 (1-T) **G**, 735 nm excitation **H**, LHCII-LL2(1-T) **I**, 735 nm excitation **J**, LHCII-HL2 (1-T) **K**, 735 nm excitation **L**, LHCII-LL1 (1-T) **M**, 735 nm excitation **N**, LHCII-HL1 (1-T) **O**, 735 nm excitation **P**. The 1-T and excitation spectrum are normalized to the fitted quantity of Chl *a* (blue and blue-green lines in all samples) in each spectrum. Spectral positions and extinction coefficients were taken from ((Croce *et al.*, 2000; Caffarri *et al.*, 2001; van den Berg *et al.*, 2018). For LHCII-LL and LhcbM1, two Loroxanthin (orange and sand colored lines), one Lutein (navy), one Violaxanthin (brown) and one Neoxanthin (turquoise) spectral forms and for LHCII-HL two Lutein (orange, and navy), one Loroxanthin (sand), one violaxanthin (brown) and one Neoxanthin (turquoise) spectral forms were used for the fitting. For ‘LhcbM1 - No Loroxanthin’, two Luteins (orange and navy) , one Violaxanthin (brown) and one Neoxanthin (turquoise) spectral forms were used for the fitting. Chl *b* spectra are colored purple and red in all samples. The spectra used for fitting were chosen based on the pigment composition determined by hplc. LHCBM1 reconstitutions from (Natali & Croce, 2015)**.** Restrictions for the fit were: 1. Individual spectral shapes and positions are the same in all 1-T and excitation spectra. 2. Individual Chl *b* and carotenoid concentrations relative to Chl *a* are similar or lower in the excitation spectrum than in the 1-T spectrum. Carotenoid EET in Table 3 is calculated by: $\frac{{integral fitted carotenoid spectra LL 735nm Exc}_{(400-520nm)}}{{integral fitted carotenoid spectra LL 1-T}_{(400-520nm)}}- \frac{{integral fitted carotenoid spectra HL 735nm Exc}_{(400-520nm)}}{{integral fitted carotenoid spectra HL 1-T}_{(400-520nm)}} \times100\%$

**
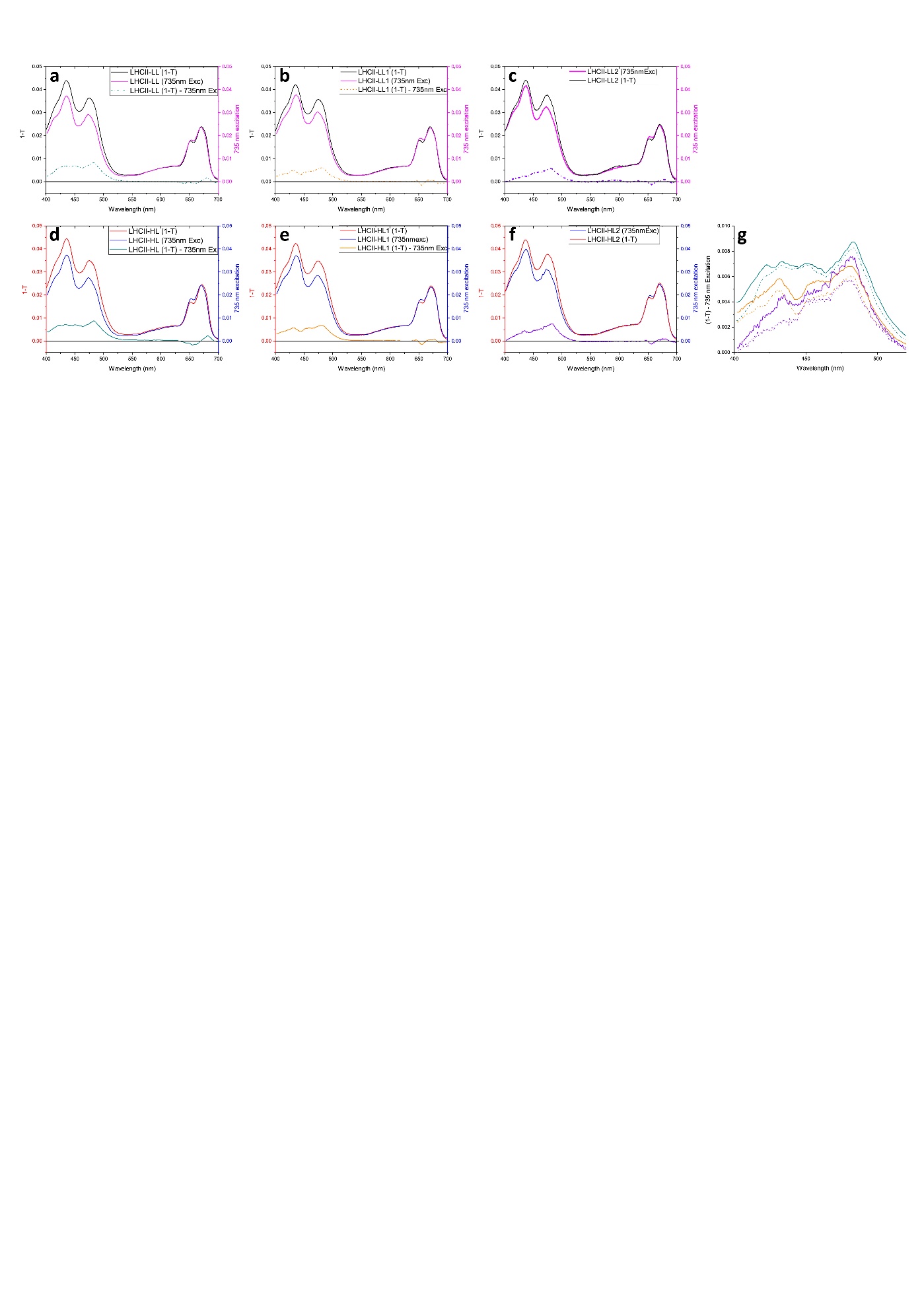
Fig. S4 Efficiency of Excitation energy transfer (EET) of LHCII-LL (three biological replicates (A,B,C) and LHCII-HL (three biological replicates (D,E,F) calculated by comparing the 1-T spectrum with the fluorescence excitation spectrum at room temperature .** Chl *a & Chl b* -> Chl *a* EET is assumed to be 100% and therefore the spectra are normalized against their integral from 620 to 700 nm. **(G)** Difference spectra ((1-T) - 735 nm excitation) of all biological replicates from A-F with similar colors, dashed LHCII-LL solid LHCII-HL. Total EET difference between LHCII-LL and LHCII-HL expressed as: $\left( \frac{\left( LL 735nm Exc \right)_{integral 400-520nm}}{\left( LL 1-T \right)_{integral 400-520nm}} \right)-\left( \frac{\left( HL 735nm Exc \right)_{integral 400-520nm}}{\left( HL 1-T \right)_{integral 400-520nm}} \right)\times100\%$, is 2.8 ± 0.4% in all biological replicate’s (P=0,019)

**
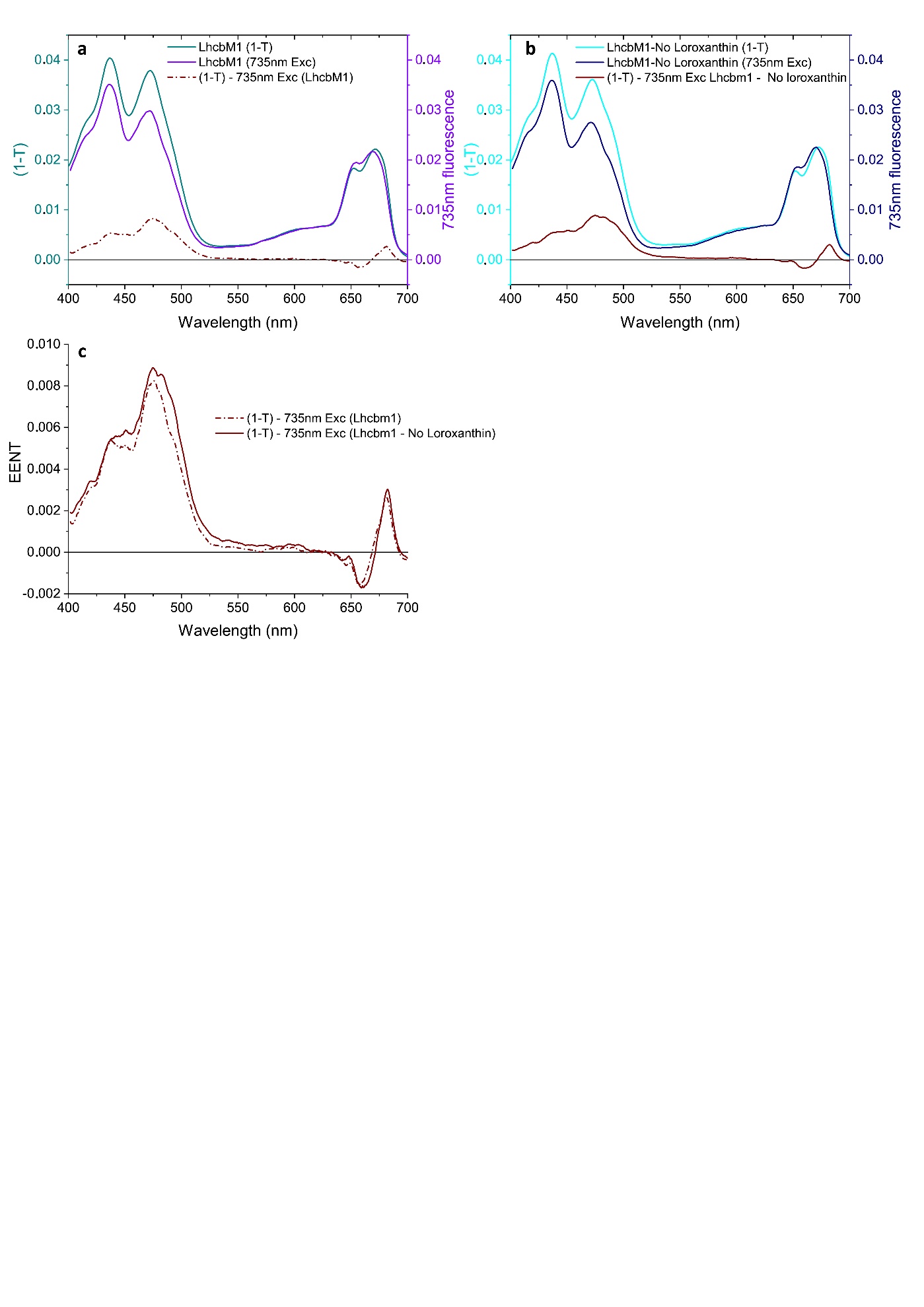
**

**Fig. S5 Efficiency of Excitation energy transfer (EET) of reconstituted monomeric LhcbM1 (A) and LhcbM1 – No Loroxanthin (B)** (Natali & Croce, 2015) **calculated by comparing the 1-T spectrum with the fluorescence excitation spectrum at room temperature.** Chl *a & Chl b* -> Chl *a* EET is assumed to be 100% and therefore the spectra are normalized against their integral from 620 to 700 nm. **(C)** Difference spectra of both samples. EENT is excitation energy not transferred. Total EET difference expressed as: $\left( \frac{\left( LHCBM1 735nm Exc \right)_{integral 400-520nm}}{\left( LHCBM1 1-T \right)_{integral 400-520nm}} \right)-\left( \frac{\left( LHCBM1-No Loroxanthin 735nm Exc \right)_{integral 400-520nm}}{\left( LHCBM1-No Loroxanthin \right)_{integral 400-520nm}} \right)\times100\%$, is 2.3%.
